# Towards an evidence-based model of fear of cancer recurrence for breast cancer survivors

**DOI:** 10.1007/s11764-016-0558-z

**Published:** 2016-07-13

**Authors:** José A. E. Custers, Marieke F. M. Gielissen, Johannes H. W. de Wilt, Aafke Honkoop, Tineke J Smilde, Dick-Johan van Spronsen, William van der Veld, Winette T. A. van der Graaf, Judith B. Prins

**Affiliations:** 1 Department of Medical Psychology, Radboud University Medical Center, PO Box 9101, 6500 HB Nijmegen, The Netherlands; 2 Department of Medical Psychology, Academic Medical Center, University of Amsterdam, Amsterdam, The Netherlands; 3 Department of Surgery, Radboud University Medical Center, Nijmegen, The Netherlands; 4 Department of Internal Medicine, Isala Klinieken, Zwolle, The Netherlands; 5 Department of Oncology, Jeroen Bosch Ziekenhuis, ‘s-Hertogenbosch, The Netherlands; 6 Department of Internal Medicine, Canisius Wilhelmina Ziekenhuis, Nijmegen, The Netherlands; 7 Department of Social Sciences, Radboud University Nijmegen, Nijmegen, The Netherlands; 8 Department of Medical Oncology, Radboud University Medical Center, Nijmegen, The Netherlands; 9 The Institute of Cancer Research and the Royal Marsden NHS Foundation Trust, London, UK

**Keywords:** Fear of cancer recurrence, Breast cancer, Psychology, Oncology

## Abstract

**Purpose:**

In order to understand the multidimensional mechanism of fear of cancer recurrence (FCR) and to identify potential targets for interventions, it is important to empirically test the theoretical model of FCR. This study aims at assessing the validity of Lee-Jones et al.’s FCR model.

**Methods:**

A total of 1205 breast cancer survivors were invited to participate in this study. Participants received a questionnaire booklet including questionnaires on demographics and psychosocial variables including FCR. Data analysis consisted of the estimation of direct and indirect effects in mediator models.

**Results:**

A total of 460 women (38 %) participated in the study. Median age was 55.8 years (range 32–87). Indirect effects of external and internal cues via FCR were found for all mediation models with limited planning for the future (*R* ^2^ = .28) and body checking (*R* ^2^ = .11–.15) as behavioral response variables, with the largest effects for limited planning for the future. A direct relation was found between feeling sick and seeking professional advice, not mediated by FCR.

**Conclusions:**

In the first tested models of FCR, all internal and external cues were associated with higher FCR. In the models with limited planning for the future and body checking as behavioral response, an indirect effect of cues via FCR was found supporting the theoretical model of Lee-Jones et al.

**Implications for Cancer Survivors:**

An evidence-based model of FCR may facilitate the development of appropriate interventions to manage FCR in breast cancer survivors.

## Introduction

With increasing breast cancer survival rates, it has become more important how survivors deal with psychosocial consequences of the disease and treatment. After curative treatment and in the absence of a physical threat, breast cancer survivors frequently have worrying thoughts about “the possibility that the cancer may return or progress in the same organ or in a different part of the body,” which is widely adopted as definition for fear of cancer recurrence (FCR) [1]. Among breast cancer survivors, FCR is commonly identified as an unmet psychosocial need for which they want help [1–6]. When FCR becomes severe, it coincides with psychological distress, lower quality of life, and functional impairments [7–9]. Since guidelines for the treatment of FCR are lacking, healthcare providers often do not know how to deal with FCR. In developing adequate care, identifying potential targets for interventions is crucial. It is therefore important to test existing theoretical models empirically to discover working mechanisms. Available models in which possible predictors are part of a multidimensional mechanism are scarce. The explanatory model of Lee-Jones is most often cited by researchers [10]. Lee-Jones and colleagues (1997) proposed a theoretical model based on Leventhal’s Self Regulation Model of Illness [11]. This self-regulation model hypothesizes that external and internal stimuli generate a subjective perception of a somatic problem or health threat and concomitant emotions (e.g., fear/distress), leading to coping strategies and appraisal of health outcomes. Representations, coping strategies, and appraisal are affected by self and social context.

Leventhal’s theoretical framework is not specific to FCR. However, it gives insight in why patients react differently to the news that they have cancer and why some are more fearful than others for cancer recurrence. Lee-Jones and colleagues developed an FCR-specific model based on Leventhal’s model, which hypothesizes that a person’s FCR will vary depending on their cognitive reaction to illness. The formulation of FCR proposed that cues play a role in activating cognitive responses associated with FCR. On the one hand, internal (somatic) cues are interpreted as reminders of the disease or as threat that the illness may have returned. On the other hand, external cues associated with the disease (e.g., medical check-ups, exposure to media) will increase worrying thoughts about a possible recurrence. High FCR can result in anxious preoccupations followed by excessive personal checking behavior, uncertainty and consequently limited planning for the future, or misinterpretation of neutral bodily symptoms and, as a consequence, excessive seeking of professional advice.

The aim of the present study was to test the hypothesized model of FCR to discover underlying mechanisms of FCR.

## Methods

### Participants

A total of 1205 eligible breast cancer survivors from three hospitals in the Netherlands (Isala Klinieken Zwolle, *n* = 461, Jeroen Bosch Hospital ‘s-Hertogenbosch, *n* = 410, and Canisius Wilhelmina Hospital Nijmegen, *n* = 334) were contacted by an information letter of their physician. It was explicitly stated in this letter that women both with and without FCR could participate. The breast cancer survivors were assessed 0–5 years after their primary treatment. To be eligible, women had to be treated with curative intent and disease-free at the time of participation. Hormonal therapy or treatment with a specific antibody (trastuzumab) was not an exclusion criterion. All participants had to be able to read and write in Dutch.

### Procedure

Documented approval from the local Ethics Committee was obtained prior to start of the study. By returning informed consent, the women agreed to participate. As part of an ongoing prospective study on the natural course of FCR, participants received a questionnaire booklet by mail or email, including questionnaires on demographic, medical, and psychosocial variables. Questionnaires could either be filled in online or in paper-and-pencil form. For the current study, baseline data were analyzed.

### Instruments

#### Cues—internal cues

##### Attention to internal bodily sensations

The Body Vigilance Scale (BVS) measures the tendency to attend to internal bodily sensations (internal cues). The BVS consists of five questions. Four questions concern the degree of attentional focus, perceived sensitivity to changes in bodily sensations, the average amount of time spent attending to sensations, and the frequency of attending to bodily sensations. The fifth question concerns the severity of 13 anxiety-related bodily sensations (heart palpitations, chest pain, numbness, tingling, shortness of breath, faintness, vision changes, dizziness, hot flash, sweating/clammy hands, upset stomach, nausea, choking/throat closing). Items are rated on a 10-point VAS [12]. In this study, Cronbach’s *α* = .91.

##### Feeling sick

Feeling sick as internal cue was assessed with a single item of the subscale Triggers of the Fear of Cancer Recurrence Inventory (FCRI): “When I feel unwell physically or when I am sick, I think about the possibility of cancer recurrence.” This item is rated on a five-point Likert scale ranging from 0 to 4. The FCRI is a reliable and valid self-report scale [13].

#### Cues—external cues

##### External cues—contact with health professionals

External cues related to contact with health professionals were measured with two items of the subscale Triggers of the FCRI: “An appointment with my doctor or other health professional” and “Medical examinations make me think about the possibility of cancer recurrence.” Both items are rated on a five-point Likert scale ranging from 0 to 4 [13]. In this study, Cronbach’s *α* = .84.

##### External cues—media and social context

External cues related to the media and social context were assessed with four items of the subscale Triggers of the FCRI: “Television shows or newspaper articles about cancer or illness,” “Conversations about cancer or illness in general,” “Seeing or hearing about someone who is ill,” and “Going to a funeral or reading the obituary section of the paper makes me think about the possibility of cancer recurrence.” Items are rated on a five-point Likert scale ranging from 0 to 4 [13]. In this study, Cronbach’s *α* = .89.

#### Fear of cancer recurrence—cognitions and emotions

##### Fear of cancer recurrence: severity

The Cancer Worry Scale (CWS) has been used in research to assess concerns about developing cancer (again) and the impact of these concerns on daily functioning. The eight items of the CWS are rated on a four-point Likert scale ranging from “Never” to “Almost always.” Scores range from 8 to 32 [14]. The CWS was validated for breast cancer survivors. A diagnostic cutoff score of 14 or higher (sensitivity 77 %; specificity 81 %) indicates elevated feelings of FCR [15]. In this study, Cronbach’s *α* = .88.

#### Consequences

##### Limited planning for the future

Limited planning for the future was assessed with one item of the subscale Functional Impairments of the FCRI: “my ability to make future plans or set life goals is disrupted by my thoughts or fears about the possibility of cancer recurrence.” Items are rated on a five-point Likert scale ranging from 0 to 4 [13].

##### Seeking professional advice

Seeking professional advice was assessed with two items of the subscale Reassurance of the FCRI. “When I think about the possibility of cancer recurrence, I use the following strategies to reassure myself: ‘I call my doctor or other health professional’, or ‘I go to the hospital or clinic for an examination.’” The items are rated on a five-point Likert scale ranging from 0 to 4 [13]. In this study, Cronbach’s *α* = .85.

##### Body checking

Body checking was assessed with a single item of the subscale Reassurance of the FCRI: “I examine myself to see if I have any physical signs of cancer.” This item is rated on a five-point Likert scale ranging from 0 to 4 [13].

### Data analyses

All analyses were performed with SPSS version 20.0. Relationships between FCR and demographic and medical variables were assessed with independent sample *t* tests and one-way ANOVAs. We estimated the model parameters with PROCESS [16]. PROCESS is a plug-in for SPSS which enables easy estimation of direct effects, indirect effects, and interactions. In addition, PROCESS estimates the significance of the indirect effect using bootstrapping which is the preferred method [17]. For simple mediation models as described here, PROCESS is comparable to other software such as Mplus, LISREL, or AMOS. In the 12 mediation analyses, the four different types of triggers were selected as independent variable and the three types of behavioral responses were selected as outcome variable. FCR was added as the mediator variable to explore and test the hypothesized models.

## Results

### Demographic and medical characteristics

Of the 1205 women who were eligible and asked to participate in this study, 565 (47 %) were interested in receiving more information about the study. Of these, 460 (38 %) women signed informed consent and returned the questionnaire booklet. Study participants were compared to 539 non-responders on age, which was the only available variable, demonstrating that participants were significantly (*t*(993,635) = 5.77, *p* < .001) younger (M = 56.69, SD = 9.6) than non-responders (M = 60.64; SD = 11.9). Data of non-responders from the Canisius Wilhelmina Hospital Nijmegen were not available. Of the women who filled out the CWS, 250 (55 %) had high FCR according to the cutoff point >13 on the CWS. Table 1 shows the patient- and treatment-related characteristics of the sample.

**Table 1:** Sample characteristics (*n* = 460)

Age (years)	Mean = 56.7, SD = 9.6; range 32–87	
Marital status: married/partnership	367	(80 %)
Children: yes	382	(83 %)
Educational level		
Primary	89	(19 %)
Secondary	224	(49 %)
Tertiary	139	(30 %)
Unknown	8	(2 %)
Employment status		
Employed	217	(47 %)
Home management	145	(32 %)
Retired	91	(20 %)
Volunteering	66	(14 %)
Sick leave	37	(8 %)
Disablement insurance act	33	(7 %)
Unemployed/others	90	(20 %)
Time since initial diagnosis (years)	Mean = 2.8; SD = 1.3	
Surgery: yes	460	(100 %)
Additional treatment		
Chemotherapy	330	(72 %)
Radiotherapy	348	(76 %)
Hormonal therapy	296	(64 %)
Trastuzumab	61	(13 %)

### Relation FCR and demographic/medical variables

With regard to demographic variables, a significant relationship was found between FCR and education (*F*(2441) = 5.26, *p* = .006), indicating that women who completed tertiary education (M = 13.78, SD = 3.6) experienced less FCR than did women who completed primary (M = 15.44, SD = 4.9) or secondary education (M = 15.00, SD = 4.2). Furthermore, there was a significant relationship between FCR and having children (*t*(445) = −2.37, *p* = .018), indicating that women with children (M = 14.94, SD = 4.3) experienced more FCR than did women without children (M = 13.66, SD = 4.0). No relationship was found between FCR and age (*r* = −.04, *p* = .415) or between FCR and partnership (*t*(438) = −.13, *p* = .899). No significant relationships were found between FCR and medical variables and between time since diagnosis (*r* = −.04, *p* = .381), chemotherapy (*t*(448) = −.11, *p* = .909), radiotherapy (*t*(447) = .55, *p* = .584), or hormonal therapy (*t*(448) = −.12, *p* = .906).

### Correlations

Table 2 displays the correlations between the measures used for testing the different models. Significant correlations ranged between .11 and .64 indicating predominantly low to moderate associations between the different variables. There was no significant relation between triggers related to contact with health professionals and seeking professional advice.

**Table 2:** Correlations between variables included in the model

	Bodily sensations BVS	Feeling sick FCRI	Media and social context FCRI	Health professionals FCRI	Limited planning FCRI	Seeking professional advice FCRI	Body checking FCRI	Fear of cancer recurrence CWS
Bodily sensations BVS	1	.392**	.322**	.296**	.155**	.143**	.305**	.352**
Feeling sick FCRI		1	.604**	.554**	.327**	.205**	.279**	.567**
Media and social context FCRI			1	.644**	.335**	.140**	.229**	.623**
Health professionals FCRI				1	.267**	.080	.246**	.522**
Limited planning FCRI					1	.114*	.225**	.529**
Seeking professional advice FCRI						1	.354**	.153**
Body checking FCRI							1	.334*
Fear of cancer recurrence CWS								1

### Mediation analysis

**Fig. 1:**
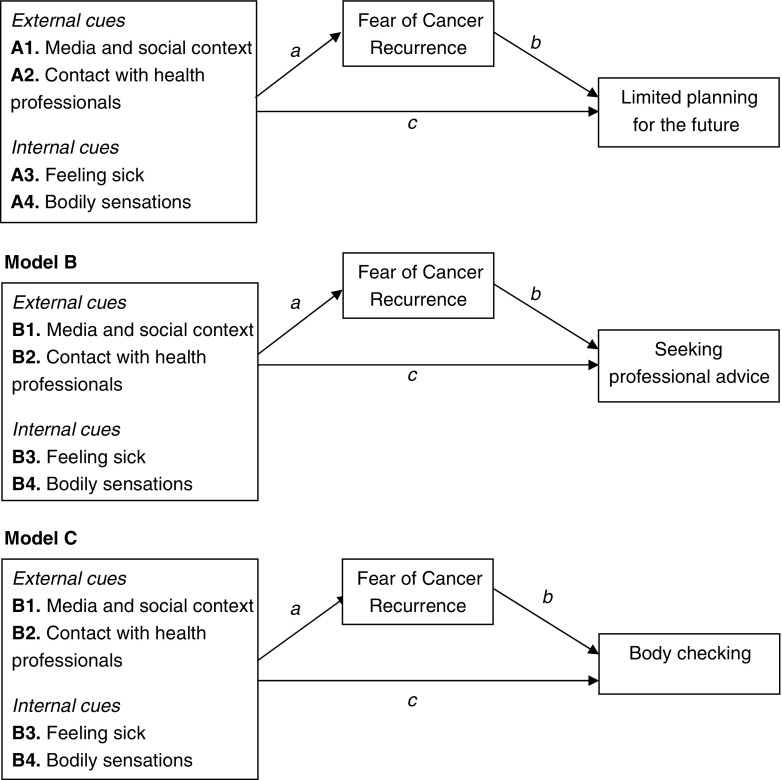
Twelve models that represent the hypotheses about the triggers and consequences of FCR

Figure 1 shows the 12 conceptual models for the associations between four types of cues (external (1 and 2), internal (3 and 4)), FCR, and three separate behavioral responses (A limited planning for the future; B seeking professional advice; C body checking). Results of the mediation analyses are displayed in Table 3. In all models, there was a positive significant relation between cues and FCR (pathway *a*). The relation between FCR and behavioral responses (pathway *b*) was significant in almost all models except for model B1 “media and social context → FCR → seeking professional advice” and model B3 “feeling sick → FCR → seeking professional advice.” In these two models, there was no direct relationship between FCR and seeking professional advice. In model B3, however, there was a significant effect of pathway *c* indicating that feeling sick was directly associated with seeking professional advice. Furthermore, significant effects of pathway *c* were found in models C3 and C4 with direct effects for feeling sick → body checking and bodily sensations → body checking. Indirect effects were found for all models with limited planning and body checking as behavioral response variable, with the largest effects for limited planning. For the models with “seeking professional advice” as outcome, the indirect effect was only significant but small for model B2 “contact with health professionals → FCR → seeking professional advice” and model B4 “bodily sensations → FCR → seeking professional advice.”

**Table 3:** Direct and indirect effects of the models

Dep. var.	Model	Trigger	*R* ^2^	*a*	*b*	*c*	Indirect effect		LLCI-ULCI^a^
Limited planning	A1	Media and social context	.278	2.64*	.128*	.000		.3397*	.2576–.4326
	A2	Contact with health professionals	.280	1.99*	.131*	−.013		.2598*	.2009–.3211
	A3	Feeling sick	.277	2.02*	.122*	.034		.2468*	.1784–.3199
	A4	Bodily sensations	.282	0.19*	.132*	−.005		.0248*	.0171–.0335
Seeking professional advice	B1	Media and social context	.026	2.64*	.021	.068		.0550	−.0262–.1323
	B2	Contact with health professionals	.023	1.99*	.031*	−.001		.0625*	.0108–.1207
	B3	Feeling sick	.043	2.02*	.012	.124*		.0243	−.0284–.0938
	B4	Bodily sensations	.031	0.19*	.025*	.010		.0047*	.0006–.0096
Body checking	C1	Media and social context	.111	2.64*	.084*	.039		.2214*	.1287–.3096
	C2	Contact with health professionals	.117	1.99*	.076*	.099		.1522*	.0918–.2186
	C3	Feeling sick	.124	2.02*	.073*	.118*		.1466*	.0876–.2190
	C4	Bodily sensations	.151	0.19*	.071*	.031*		.0133*	.0071–.0197

^a^Lower and upper limits of the bootstrap confidence interval of the indirect effect

*p < .05

## Discussion

To the best of our knowledge, this is the first study to test the hypothesized model of Lee-Jones [10]. The results showed evidence for almost all relationships in the model except for the indirect effect with seeking professional advice as behavioral response. The models with limited planning for the future as behavioral consequence showed the largest effects. In the model with seeking professional advice as behavioral response, only very small indirect effects were found for two types of cues (external triggers related to oneself; internal bodily sensations). Furthermore, there was a direct relation between feeling sick and seeking professional advice and a marginal significant direct relation between bodily sensations and seeking professional advice. These effects point in the direction that when patients experience symptoms they directly contact their health professional for advice. However, since the “feeling sick” item specifically refers to FCR in the questionnaire, increased FCR could still play a role in the relationship between symptoms and seeking professional advice. In the mediation models with bodily symptoms as cues, the indirect effects were small. This could probably be explained by the fact that the bodily symptom items focused on the tendency to be hypervigilant to internal signals instead of the occurrence of internal triggers. Furthermore, the other types of cues and behavioral responses were derived from the FCRI which focused specifically on FCR, thus inflating the chance to find significant associations with FCR.

With regard to the relationship between FCR and demographic or medical variables, only lower education and having children were associated with higher FCR. Although younger age is commonly reported in literature reviews [7–9] to be associated with higher FCR, we did not find a relationship in this study. However, study participants were significantly younger than non-respondents. Since participants were aware of the fact that the purpose of the study was FCR, it is plausible that there was already a selection in signups for the study as confirmed by the moderate response rate of 38 % and the relatively high percentage of 55 % highly fearful survivors. Selection bias is an aspect that should be taken into account when designing research on FCR since a proportion of survivors recognize their FCR and express a need for help whereas other survivors cope with FCR by avoiding threat, including study questionnaires.

A limitation of this study is the cross-sectional design, which makes it difficult to draw conclusions about causality. Although the basis of this research was a strong theoretical model proposed in the literature, future research should focus on the direction of the relationships and the possibility of bidirectional relationships within the model to further strengthen our theoretical knowledge of FCR. Furthermore, the use of single items derived from subscales of the FCRI might distort the results since psychometric properties of these items are not known. Also, the items of the FCRI contained already an element of cognitive interpretation related to FCR where the actual presence of cues would be more accurate. To disentangle the actual presence of cues from a reaction or interpretation of these cues, questionnaires assessing the frequency of cancer-related somatic symptoms that are less prone to be influenced by cognitive interpretation would have been more appropriate. Therefore, in this study, it is not yet clear whether the results solely support the validity of the model or indicate the extent to which the constructs share a common cognitive component. It would be interesting for future research to see if the results obtained in this sample of breast cancer survivors could be generalized to other types of cancer. Furthermore, the starting base of this study was to explore the mechanism of FCR along the continuum from low to high FCR. To see whether predictors of clinical levels of FCR are different from predictors of FCR in general would be a next step. The results of this study might have future implications and suggestions for different professions. For clinicians, it is important to keep in mind that patients are very aware of bodily symptoms and that FCR is also triggered by information about cancer in their surroundings. Clinicians may provide tailored and correct information about one’s disease status and education about symptoms that require immediate action versus symptoms that might be innocent. Once they received this information, patients might be better able to reassure themselves and adopt a wait-and-see approach. For psychologists, the model confirms that a cognitive behavioral therapeutic approach might be a good intervention for high FCR. More specifically, the results of this study indicate that interventions targeting FCR may benefit from incorporating modules on making new plans for the future and the regulation of bodily checking behaviors. Interventions including these elements are currently being tested [18, 19]. For researchers, this study gives insight into how FCR model testing can be performed. Replication of this study in a longitudinal design is needed, taking into account some methodological considerations about the best questionnaires to use for risk factors and protective factors. A greater understanding of these factors associated with FCR may assist the development of evidence-based screening programs and treatments for FCR.
